# Genetic Parentage Analysis Confirms a Polygynandrous Breeding System in the European Grayling (*Thymallus thymallus*)

**DOI:** 10.1371/journal.pone.0122032

**Published:** 2015-03-20

**Authors:** Peter Jørgen Haddeland, Claudia Junge, Dimitar Serbezov, Leif Asbjørn Vøllestad

**Affiliations:** 1 Center for Ecological and Evolutionary Synthesis, Department of Biosciences, University of Oslo, P. O. Box 1066, Blindern, 0316, Oslo, Norway; 2 Department of Genetic Kinship and Identity, Norwegian Institute of Public Health, P. O. Box 4040, Nydalen, 0403, Oslo, Norway; 3 Southern Seas Ecology Laboratories, DP418, School of Biological Sciences, University of Adelaide, Adelaide, SA 5005, Australia; 4 National Agency of Fisheries and Aquaculture, 17 Hristo Botev Blvd, 1606, Sofia, Bulgaria; CNRS, FRANCE

## Abstract

Knowing the breeding system of a species is important in order to understand individual variation in reproductive success. Large variation in reproductive success and thus reproductive skew strongly impacts on the effective number of breeders and thus the long-term effective population size (N_e_). Fishes, in particular species belonging to the salmonid family, exhibit a wide diversity of breeding systems. In general, however, breeding systems are rarely studied in detail in the wild. Here we examine the breeding system of the spring-spawning European grayling *Thymallus thymallus* from a small Norwegian stream using parentage assignment based on the genotyping of 19 polymorphic microsatellite loci. In total 895 individual grayling fry and 154 mature grayling (57 females and 97 males) were genotyped. A total of 466 offspring were assigned a father, a mother, or a parent pair with a confidence of 90% or higher. Successfully reproducing males had on average 11.9 ± 13.3 (SD) offspring with on average 2.1 ± 1.2 partners, whereas successful females had on average 9.5 ± 12.8 offspring and 2.3 ± 1.5 partners. Parents with more partners also produced more offspring. Thus the grayling breeding system within this small stream revealed a polygynandrous breeding system, similar to what has been observed for many other salmonid fish species. The present study thus unambiguously corroborates a polygynadrous breeding system in the European grayling. This knowledge is critical for managing populations of this species, which has suffered significant local population declines throughout its range over the last several decades.

## Introduction

Animals have a wide variety of breeding systems, ranging from promiscuous to monogamous [1,2]. For many species the breeding system is more or less unknown. The breeding system of a species impacts on the variation in individual reproductive success and reproductive skew. Among-individual variation in breeding success may thus lead to mate-mate competition and mate choice [3]. The level of reproductive skew furthermore strongly impacts on the effective number of breeders and thus the long-term effective population size (N_e_) [4,5] which highlights the need for a detailed understanding of a species’ breeding system, especially for species of conservation concern.

Fishes in general [2,6], and salmonid fishes in particular, exhibit a wide diversity of breeding systems [7,8]. Even within a population, both female and male reproductive success may vary considerably [8–10]. Salmonids have aggregate breeding systems and competition for mates can be intense. Differential reproductive success and thus reproductive skew is a probable outcome. This variation creates opportunity for sexual selection that might shape their behaviour, morphology and life history.

It has, however, in the past been difficult to study the breeding system of salmonid fish species in detail as their fertilisation is external and accurate observations under water are difficult to attain. Recently, however, the availability of genetic genotyping and parentage assignment methods has lead to a number of studies on the genetic breeding system of salmonid fishes [9–11]. Based on these studies it seems like the ‘typical’ salmonid breeding system is polygamous or polygynandrous. Males compete for access to receptive females and females choose among males [8,12,13]. Salmonid breeding systems vary in the degrees of intra-sex competition, strength of sexual selection in the two sexes, and the development of secondary sexual characteristics. Reproductive skew is therefore to be expected, and has been documented in a number of recent studies using molecular genotyping techniques and parentage assignment performed in the wild [10,14,15].

The European grayling *Thymallus thymallus* is a spring-spawning freshwater salmonid for which little is known about its breeding system. Grayling exhibit iteroparity (repeat spawning), and earlier observational studies indicate that they have a polygynandrous breeding system where both sexes mate with more than one partner within the same spawning season [16–19]. A recent study using parental assignment of a limited number of larvae do indicate that the mating system is polygynandrous [20]. Males guard against other males a spawning territory where they are approached by mature female grayling. Dominant males may therefore hold high-quality territories and attract more females. Larger males would then be expected to have higher individual reproductive success. Also, as female fecundity increases with female body size [21], larger females are expected to have higher individual reproductive success than smaller individuals.

The overall aim of this study was to investigate the genetic breeding system in a small stream-spawning grayling population by testing if grayling indeed exhibit a polygynandrous breeding system, and investigating the level of variation in individual reproductive success and potential causes for such individual differences in both sexes.

## Material and Methods

### Study species

Grayling is a salmonid fish that usually spawns in small rivers and streams during spring or early summer [18]. Male grayling arrive early on the spawning ground and are aggressive, attacking both trespassing males and unripe females, thus larger males usually acquire better spawning sites [16]. The eggs hatch after 140–200 degree-days, and the juveniles become free-swimming after another ca. 140 degree-days [22–25]. After the larvae emerge from the gravel and become free-living, they might either stay in the tributary for some time before they migrate or drift downstream shortly after emergence [22,26,27].

### Study site and sampling

Lake Lesjaskogsvatnet is a shallow (mean depth of 10 metres) mountain lake (611 meters above sea level) with a surface area of approximately 4.52 km^2^. Grayling were introduced to the Lesjaskogsvatnet system at the end of the 19^th^ century [22]. Since then spawning populations have been established in a large number of tributaries. The populations are now weakly genetically differentiated based on neutral genetic markers [28,29]. Despite the weak structuring the populations differ in a number of genetically based early life history traits [25,30] as well as in gene expression [31]. Søre Skottåe is one of the tributaries used by grayling in Lesjaskogsvatnet. It is a small stream, 1–1.5 meters wide, in the northeast end of the lake [32]. Each year grayling ascend the stream for spawning during a few days in May-June. The exact timing of spawning depends on local environmental conditions that vary strongly among years [33].

Migrating adult grayling were sampled with fyke nets in Søre Skottåe during the spawning season 2008. In total 149 mature grayling were captured during a 10-day period starting on May 31^st^ and ending on June 11^th^. Very few fish ascended the stream after June 7^th^. This indicates that migration and spawning is highly synchronized. The effective population size (N_e_) of the grayling in this tributary has earlier been estimated to 63 (confidence interval 40–126) [28], based on samples collected in 2001 and 2008. N_e_ in that study was estimated based on short-term allelic frequency changes using a method allowing for migration [34]. Thus, the Søre Skottåe grayling population is relatively small.

All captured mature fish were anesthetized with clove oil [35], their fork length was measured (nearest mm) and they were sexed based on external sexual characters. Further, the adipose fin was excised and stored in 96% ethanol for later genetic analysis. The individuals were allowed to recover from the anaesthesia before they were released back to the stream upstream of the nets to complete their spawning.

Grayling fry (n = 895) were subsequently sampled with drift nets over a period of approximately three weeks in July 2008. A number of drift nets were deployed to cover most of the stream to collect larvae drifting downstream with the current. The drift net also collected large amounts of debris so that the fry had to be sorted from the debris afterwards. Sometimes individual fry were crushed while in the nets; if that was the case all potential pieces were collected (and separated into individuals based on genotypes later). The majority (>90%) of the fry were caught during two days (9^th^ and 10^th^ July) indicating that drift happens over a very short time period. We thus assume that the acquired sample is a random representation of all downstream drifting fry. The fry were stored individually in 96% ethanol until later DNA isolation.

### Ethics statement

Animal sampling and experimentation were performed in compliance with permission given by the Norwegian Animal Research Authority (permission ID 2008/7368.5). All mature fish were returned to the stream to complete natural reproduction after tissue sampling. The grayling is listed as a species of least concern (LC) on the Norwegian Red List (2010).

### Genotyping

DNA was extracted using the salt extraction method [36], or by the DNeasy Blood & Tissue Kit (Qiagen, Hilden, Germany) according to manufacturer’s protocol.

The polymerase chain reaction (PCR) amplifications of the 19 polymorphic microsatellite loci were performed in seven different reactions, two single and five multiplex PCRs (Table 1) [28,37]. In short, the PCRs had annealing temperatures ranging from 58°C to 60°C and each individual reaction consisted of 2x Qiagen multiplex PCR master mix, 1.5 μl of DNA, primer concentration varying from 0.04–057 μM, and sterile H_2_O. PCR cycles were: 95°C for 15 min; followed by 37 cycles of: 94°C for 30 s, 58–60°C for 1 min and 30 sec, 72°C for 1 min; then 60°C for 30 min. All PCR products from one individual were combined, diluted and 2 μl of those were added to a 10:1 mix of formamide and GeneScan—600 LIZ size standard (Applied Biosystems (ABI), CA, USA), reaching a total volume of 12 μl in each well. This was then electrophoresed on an ABI 3730 DNA analyser (ABI) and genotypes were scored using GeneMapper 4.0 software (ABI). Positive controls were included on a regular basis and all the scored alleles were visually checked after the automated scoring process to minimize the amount of scoring errors and maximize accuracy.

**Table 1:** Microsatellite loci and primers used in the study.

*Locus name*	*Multiplex group*	*Dye*	*Primer Concentration (μM)*	*Annealing temperature*	*Na*	*Allelic range*
BFRO13^§^	MP1	FAM	0.09	58	4	235–247
213^#^	single	FAM	0.38	60	11	283–327
414^#^	MP2	FAM	0.20	60	6	393–413
309^#^	MP4	FAM	0.57	59	2	447–451
TAR106^$^	MP5	FAM	0.07	59	8	193–221
BFRO10**	MP1	VIC	0.08	58	2	96–122
BFRO15^&^	MP1	VIC	0.04	58	2	144–154
BFRO18^&^	MP1	VIC	0.04	58	4	181–195
207^#^	MP1	VIC	0.10	58	2	216–224
BFRO9*	MP1	VIC	0.05	58	2	243–247
438^#^	single	VIC	0.34	60	9	265–297
BFRO11**	MP3	NED	0.30	59	2	86–102
313^#^	MP2	NED	0.18	60	6	180–200
Ogo2^@^	MP1	NED	0.07	58	3	233–241
433b^#^	MP3	NED	0.18	59	8	287–315
445^#^	MP4	NED	0.13	59	12	374–422
415^#^	MP3	PET	0.33	59	9	193–225
214^#^	MP1	PET	0.14	58	4	292–313
407b^#^	MP5	PET	0.20	59	7	230–254

The multiplex group for the locus or if it was single locus PCR amplification is given as well as primer concentration, fluorescent dye type, annealing temperature, number of alleles (Na) and allelic range (base pairs).

^§^ GenBank: AF151370,

^#^ [53],

^$^ [37],

* [54],

^&^ [55],

** [56],

^@^ [57]

### Analyses

GenAlEx 6.41 [38] was used to perform a multi-locus match analysis for co-dominant data to ensure that no individual grayling appeared more than once in the data set. This was particularly important in this study as some of the fry were retrieved only as pieces from the drift nets, since the small delicate fry easily get damaged when captured together with different kinds of floating debris. CERVUS 3.0 [39,40] was used to calculate the polymorphic information content (PIC) of each of the 19 microsatellite loci. The PIC value of a locus is calculated from the allele frequencies, and the PIC is a measure of the information content and variation at each locus. PIC values ranging from 0–0.29 is considered uninformative, from 0.3–0.59 is considered moderately informative, and a PIC above 0.6 is considered highly informative [41]. GenAlEx was further used to calculate the observed (H_O_) and unbiased expected (H_E_) heterozygosity and to check for potential deviations from Hardy-Weinberg equilibrium (HWE) for the sample of adult fish.

We used a Bayesian approach implemented in the R package MasterBayes [42]. Using Markov Chain Monte Carlo (MCMC) methods, MasterBayes confidence in parentage is assessed at the level of individual assignments using all the information provided by potential parents. The pedigree configuration was very similar regardless of the priors used, a testimony of the adequacy of the data used for such analyses. The default uniform priors were consequently used. The Markov chains converged easily, and runs were performed with 1300000 iterations, burn in interval of 300000 iterations, and a thinning interval of 100. Maximum one mismatch between parent and offspring genotypes was allowed when performing the assignments. Here, we use a cut-off for assignment probability of 90%.

There are a number of different software for performing parentage assignments, each with different assumptions and algorithms. As a test of the assignment procedure we also used COLONY v. 2.0 [43,44] to assign progeny into half- and full-sib families with known parents. These results were only used as a comparison of methods; the main analysis is based on the MasterBayes assignments.

We used the parentage assignment results to get an estimate for the effective number of breeders (N_b_) in this reproductive event. The effective number of female breeders, N_bf_, was calculated using N_bf_ = k_f_ * (N_f_ - 1)/(1 + V_f_/k_f_), where N_f_ is the number of sexually mature females and k_f_ and V_kf_ are the mean and the variance of the number of progeny produced [45]. The effective number of male breeders, N_bm_, was calculated analogously. The total effective number of breeders was then calculated as N_b_ = 4* (N_bf_ * N_bm_)/ (N_bf_ + N_bm_). To obtain confidence intervals for the estimated N_b_, we resampled with replacement the reproductive success (number of offspring) of the observed spawners. This bootstrap procedure was repeated 1000 times, and the 2.5% and 97% percentiles were taken as the lower and upper 95% confidence limits for N_b_, respectively.

Generalized linear models (GLM) were used to test for relationships between individual reproductive success (number of assigned progeny, number of partners) and fork length and for the relationship between number of partners and number of assigned progeny. A Poisson distribution with a log-link was used, implemented in JMP [46].

## Results

In total 895 individual grayling fry samples and fin clips from 154 mature grayling (57 females and 97 males) were collected. The male grayling were significantly larger (mean fork length ± SD; 321 ± 28 mm) than female grayling (288 ± 19 mm; Welch two sample t-test, t = -8.62, p < 0.001). Of the sampled and genotyped fry samples, multi-locus match analysis for co-dominant data in GenAlEx revealed that 45 samples had to be removed as their exact genotype already appeared in the data set (probability of identity over 19 loci = 7.5E-14). This was due to some fry samples being composed of small bits of tissue due to interaction with all the debris that was collected together with the fry in the drift traps. Consequently, 840 unique fry genotypes were analysed in this study. The mean total length of a subset of 530 fry (60% of the total sample) was 13.9 ± 1.5 mm, ranging from 8 to 17 mm.

The mean polymorphic information content (PIC) over all loci ranged from 0.15 to 0.82 (mean ± SD, 0.53 ± 0.19) (Table 1). The 19 loci were moderately to highly informative (2 loci below 0.3, 9 loci 0.3–0.59, 8 loci above 0.6). The mean unbiased expected heterozygosity for the adults was 0.59, and the mean observed heterozygosity was 0.60 (Table 2). The adult genotypes deviated from Hardy-Weinberg equilibrium at only one locus (Tth-407b), which is not significant after adjustment for multiple tests. This indicates that the sample of mature fish can be considered a random sample of the spawning population.

**Table 2:** Genetic diversity indices for all 19 loci for the adult grayling.

*Locus*	*H*_*E*_	*H*_*O*_	*H-W*	*PIC*
BFRO13	0.65	0.70	0.445	0.59
213	0.79	0.84	0.204	0.76
414	0.73	0.71	0.919	0.68
309	0.45	0.40	0.180	0.35
TAR106	0.67	0.64	0.588	0.62
BFRO10	0.36	0.36	0.852	0.29
BFRO15	0.50	0.51	0.744	0.38
BFRO18	0.55	0.59	0.644	0.47
207	0.49	0.52	0.561	0.36
BFRO9	0.17	0.17	0.790	0.15
438	0.80	0.83	0.909	0.77
BFRO11	0.45	0.46	0.658	0.35
313	0.74	0.77	0.909	0.69
Ogo2	0.64	0.68	0.737	0.56
433b	0.66	0.60	0.469	0.61
445	0.84	0.86	0.936	0.82
415	0.76	0.77	0.714	0.72
214	0.50	0.51	0.241	0.45
407b	0.44	0.39	**0.005**	0.42
*Mean±se*	*0*.*59±0*.*4*	*0*.*60±0*.*04*	*-*	*0*.*53±0*.*19*

H_E_ is the unbiased expected heterozygosity, H_O_ is the observed heterozygosity, H-W states the significance test (p-value) for deviation from Hardy-Weinberg equilibrium (deviating loci are in bold) and PIC is the polymorphic information content of each locus. The last row contains the average and the standard deviation of the heterozygosity and PIC across all loci.

MasterBayes assigned a total of 466 offspring with confidence of 90% or higher. Of these 346 offspring were assigned to a sampled father, 203 offspring were assigned to a sampled mother and 149 offspring were assigned to both a sampled mother and a sampled father. A total of 32 males out of 97 (33.0%) were assigned offspring, whereas 22 out of 57 (38.6%) females were assigned offspring.

The parentage assignments based on COLONY were very similar to that based on MasterBayes. Around 90% of the individuals that were assigned parentage with COLONY were also assigned parentage with MasterBayes (91.0% for fathers, 89.2% for mothers).

**Fig 1:**
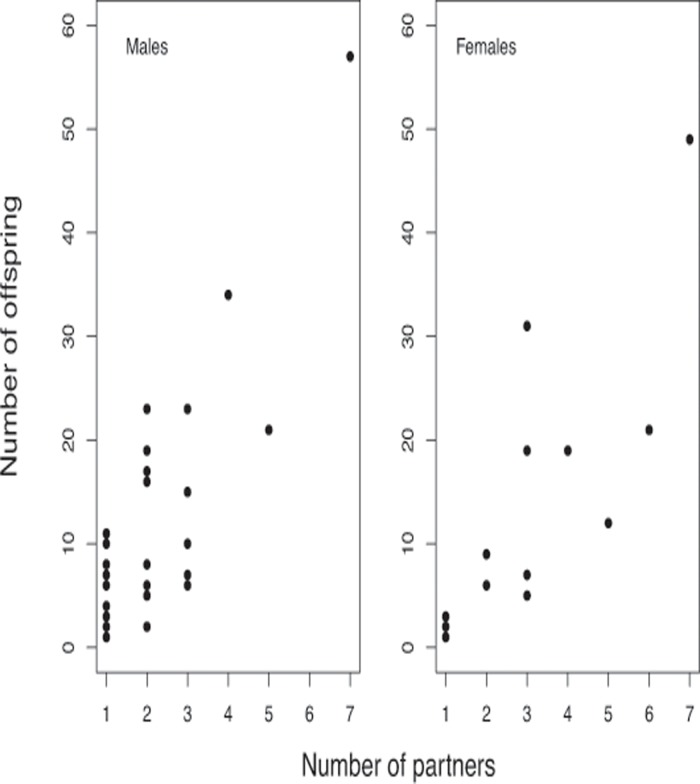
Number of breeding partners. Estimated number of partners and offspring for male and female grayling breeding pairs.

**Fig 2:**
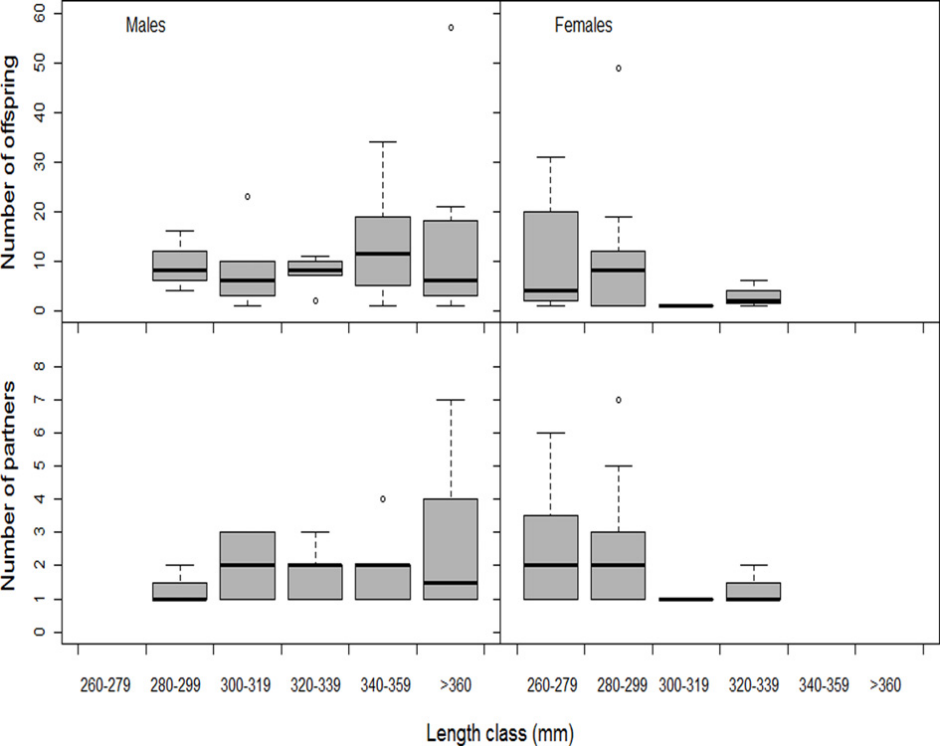
Effect of grayling length (mm) of assigned number of partners and offspring. Data are presented as box plots for each 20 mm length bin.

Many of the progeny were only assigned (using MasterBayes) either a father or a mother. When evaluating the number of partners of each individual, we assumed that progeny having the same mother or father, but with an unassigned parent, was parented by only one individual of the opposite gender. Successfully reproducing males had on average 11.9 ± 13.3 (SD) (range 1–62) offspring with on average 2.1 ± 1.2 (range 1–6) partners, and males with more partners also sired more offspring (generalized linear model with Poisson distribution and log-link, χ^2^ = 249.0, n = 35, P < 0.0001) (Fig. 1). The successful females had on average 9.5 ± 12.8 (range 1–51) offspring with on average 2.3 ± 1.5 (range 1–6) partners, and females with more partners also sired more offspring (GLM, χ^2^ = 205.0, n = 25, P < 0.0001; Fig. 1). Larger males produced more progeny (GLM, χ^2^ = 36.8, n = 35, P < 0.0001) but they did not have more partners (GLM, χ^2^ = 1.35, n = 35, P = 0.250) (Fig. 2). For the females, there was a tendency for larger females to produce fewer progeny (χ^2^ = 5.6, n = 25, P = 0.017), and there was no relationship between female size and number of partners (GLM, χ^2^ = 0.001, n = 35, P = 0.980; Fig. 2).

Based on the family size variation, the effective number of breeders (N_b_) in the population was estimated to be 24.7 (96% confidence interval: 16.1–35.5).

## Discussion

The genetic investigation of the European grayling breeding system within this small alpine stream revealed a polygynandrous breeding system. This has previously been suggested on the basis of observational studies of behaviour [16,17,19]. Further, in a recent study Meraner et al. [20] used sibship reconstruction methods to show that a sample of 22 related juveniles identified in their study was aggregated into four full-sib families nested into 8 paternal and 5 maternal half-sib family groups. In our study we found strong evidence that many males and females successfully produced offspring with more than one partner, and that the individual reproductive success was highly skewed. Both for males and females it was clear that the individuals with more partners also produce more progeny. In total, this should open for strong sexual selection in the system.

There was large variation in individual reproductive success, both for male and female grayling. Generally, male reproductive success in salmonid fishes such as salmon, trout and grayling will be determined by access (proximity) to females during the actual spawning whereas female reproductive success mainly is assumed to be determined by fecundity [12]. The fecundity of female grayling is positively related to female size [18,21]. Larger females have the potential to produce more eggs and thus potentially have more offspring than the smaller females. This was, however, not the case for the grayling in this study; actually there was a weak tendency for the larger females to produce somewhat fewer progeny. In most other studies of the breeding system of salmonid fishes no or a weakly positive relationship between female size and reproductive success have been found [9,10,14,47]. Thus, other factors such as male quality and the quality of the selected spawning habitat might be more important for female fitness. Further, in the grayling population studied here the variation in female size was relatively limited, both leading to relatively small differences in expected fecundity and to low power of detecting a size-effect if such an effect was present.

The larger grayling males sired more offspring than smaller males. For male salmonids it is usually assumed that large males, due to their dominance and competitive ability, will get the larger amount of breeding attempts and thus higher reproductive success [12]. However, other studies of the breeding system of salmonid fishes have produced conflicting results [9,10,14,48], indicating that other factors than size may be just as important for success. As mentioned above, habitat quality may be one such factor. Timing of spawning may also impact on reproductive success, especially when there is an extended duration of the spawning season. In our study system all grayling migrated into the stream during a very short time window, and based on experience with this and other grayling systems the actual spawning happens soon after ascent into the streams. Given this very limited variance in timing of ascent it was not possible to test for any effect of timing on success.

The number of partners differed strongly among individual grayling. Overall, the number of partners for the successful individuals varied from 1 to 6, with an average of approximately 2 partners for both males and females. However, most of the spawners were not assigned a partner and were thus unsuccessful. It is suggested that males have larger opportunity than females to mate multiple times as individual males may be sexually active for longer time periods than females. Females, on the other hand, have a short time window following ovulation when spawning has to be completed. In total this may lead to a male-biased operational sex ratio (OSR, the ratio of sexually active females to males [49]). In our study we also observed a larger number of males than females, enhancing the possibility for a male-based OSR. Such a male-biased sex ratio was described recently for grayling in Lake Thun, Switzerland [50]. Such male biased operational sex ratios may lead to strong male-male competition, allow for female choice and lead to strong intra-sexual selection [12]. Dominant males thus have the opportunity to acquire multiple mates, potentially also producing a strong male reproductive skew [9,10,14]. However, the numbers of partners for successful male and female grayling were very similar. One reason for this may be that the spawning season for the grayling is very short in this stream (see also [33]). Grayling usually spawn at temperatures between 5–8°C [51,52] and during spring the small stream studied here rapidly becomes warmer than this. This reduces the time available for males to acquire multiple mates since receptive females are only available during a relatively short time interval. Such a short spawning period would also lessen the potential for strong sexual selection.

The grayling population studied here is relatively small, with an observed number of spawners (N) of 154. It is possible that the population is larger, as sampling of migrating fish was not 100% efficient at all times. However, the population effective number of breeders estimated based on the family size variances is also small (N_b_ = 24.7). In a previous study based on data from 2001 and 2008 (this sample), the effective population size was estimated using the temporal method (MNe 1.0; [34]) to be 63 (CI: 40–126) [28]. Therefore, although the actual number of breeders probably is higher than the number of effective breeders, it is still unlikely that the Søre Skottåe grayling represent a large population. Overall, these estimates indicated an Nb/N ratio of 0.16 and a Ne/N ratio of 0.41. This is very similarly to what we recently observed in an extensively studied brown trout *Salmo trutta* population (see estimates and discussion in [4]).
